# Age- and Sex-Dependent Patterns of Gut Microbial Diversity in Human Adults

**DOI:** 10.1128/mSystems.00261-19

**Published:** 2019-05-14

**Authors:** Jacobo de la Cuesta-Zuluaga, Scott T. Kelley, Yingfeng Chen, Juan S. Escobar, Noel T. Mueller, Ruth E. Ley, Daniel McDonald, Shi Huang, Austin D. Swafford, Rob Knight, Varykina G. Thackray

**Affiliations:** a Department of Microbiome Science, Max Planck Institute for Developmental Biology, Tübingen, Germany; b Department of Biology, San Diego State University, San Diego, California, USA; c Vidarium—Nutrition, Health and Wellness Research Center, Grupo Empresarial Nutresa, Medellin, Colombia; d Department of Epidemiology, Johns Hopkins Bloomberg School of Public Health, Baltimore, Maryland, USA; e Welch Center for Epidemiology, Prevention and Clinical Research, Johns Hopkins Medical Institutions, Baltimore, Maryland, USA; f Department of Pediatrics, University of California, San Diego, La Jolla, California, USA; g Center for Microbiome Innovation, University of California, San Diego, La Jolla, California, USA; h Department of Computer Science, University of California, San Diego, La Jolla, California, USA; i Department of Bioengineering, University of California, San Diego, La Jolla, California, USA; j Department of Obstetrics, Gynecology and Reproductive Sciences, University of California, San Diego, La Jolla, California, USA; University of California, San Francisco

**Keywords:** 16S rRNA amplicon, age, diversity, microbiome, sex

## Abstract

Gut microbial diversity changes throughout the human life span and is known to be associated with host sex. We investigated the association of age, sex, and gut bacterial alpha diversity in three large cohorts of adults from four geographical regions: subjects from the United States and United Kingdom in the American Gut Project (AGP) citizen-science initiative and two independent cohorts of Colombians and Chinese. In three of the four cohorts, we observed a strong positive association between age and alpha diversity in young adults that plateaued after age 40 years. We also found sex-dependent differences that were more pronounced in younger adults than in middle-aged adults, with women having higher alpha diversity than men. In contrast to the other three cohorts, no association of alpha diversity with age or sex was observed in the Chinese cohort. The association of alpha diversity with age and sex remained after adjusting for cardiometabolic parameters in the Colombian cohort and antibiotic usage in the AGP cohort. We further attempted to predict the microbiota age in individuals using a machine-learning approach for the men and women in each cohort. Consistent with our alpha-diversity-based findings, U.S. and U.K. women had a significantly higher predicted microbiota age than men, with a reduced difference being seen above age 40 years. This difference was not observed in the Colombian cohort and was observed only in middle-aged Chinese adults. Together, our results provide new insights into the influence of age and sex on the biodiversity of the human gut microbiota during adulthood while highlighting similarities and differences across diverse cohorts.

**IMPORTANCE** Microorganisms in the human gut play a role in health and disease, and in adults higher gut biodiversity has been linked to better health. Since gut microorganisms may be pivotal in the development of microbial therapies, understanding the factors that shape gut biodiversity is of utmost interest. We performed large-scale analyses of the relationship of age and sex to gut bacterial diversity in adult cohorts from four geographic regions: the United States, the United Kingdom, Colombia, and China. In the U.S., U.K., and Colombian cohorts, bacterial biodiversity correlated positively with age in young adults but plateaued at about age 40 years, with no positive association being found in middle-aged adults. Young, but not middle-aged, adult women had higher gut bacterial diversity than men, a pattern confirmed via supervised machine learning. Interestingly, in the Chinese cohort, minimal associations were observed between gut biodiversity and age or sex. Our results highlight the patterns of adult gut biodiversity and provide a framework for future research.

## Abstract (precis)

Microorganisms in the human gut play a role in health and disease, and in adults higher gut biodiversity has been linked to better health. Since gut microorganisms may be pivotal in the development of microbial therapies, understanding the factors that shape gut biodiversity is of utmost interest. We performed large-scale analyses of the relationship of age and sex to gut bacterial diversity in adult cohorts from four geographic regions: the United States, the United Kingdom, Colombia, and China. In the U.S., U.K., and Colombian cohorts, bacterial biodiversity correlated positively with age in young adults but plateaued at about age 40 years, with no positive association being found in middle-aged adults. Young, but not middle-aged, adult women had higher gut bacterial diversity than men, a pattern confirmed via supervised machine learning. Interestingly, in the Chinese cohort, minimal associations were observed between gut biodiversity and age or sex. Our results highlight the patterns of adult gut biodiversity and provide a framework for future research.

## INTRODUCTION

The human gut microbiota is a highly diverse ecosystem that is extremely variable among individuals (1). This microbial community may play a key role in human health and disease (2). Since the gut microbiota may be pivotal to the development of microbial therapies, understanding the factors that shape overall gut microbiota biodiversity over the different human life stages is of utmost interest.

There is increasing evidence suggesting that host genes, gene expression patterns, environmental exposures (including medication and diet), and lifestyle factors play an important role in delimiting the boundaries of microbial diversity in the gut (3, 4). While a detailed longitudinal study of the interplay of each of these factors would be scientifically, logistically, and financially challenging, the chronological age of the host may be conceived of as a proxy variable that represents the accumulation of these effects for a given individual. Several studies have reported a positive correlation between age and gut microbiota alpha diversity from birth to adulthood (5–8). Likewise, it has been shown that alpha diversity is maintained in old age, until comorbidities contribute to its decline (9). Another intriguing host-associated pattern identified in humans and rodents is the link between the gut microbiota and biological sex. Several studies have reported that women have higher microbial diversity than men and that sex differences in microbial composition emerge after puberty (8, 10–13). These differences may contribute to the sexual dimorphism of autoimmune (12, 14, 15) and neuroimmune (16, 17) diseases. Therefore, it is key to consider the impact of age and sex differences in different human populations to adequately discriminate changes and variations in the microbiome of individuals.

To better understand how the age and sex of the host relate to the diversity of the gut microbiota during adulthood, we explored the association of these factors using data from individuals in three cross-sectional studies from four geographical origins, including the citizen-science American Gut Project (AGP), comprised of individuals from the United States and the United Kingdom (4); a cohort of individuals from China (18); and a study of community-dwelling adults from Colombia (19).

## RESULTS

The basic characteristics of the individuals from the four cohorts, stratified by sex and age group, are summarized in Table 1. We defined adults as individuals between 20 and 69 years of age and divided the age groups by the middle point of this range (i.e., 45 years); subjects above 70 years of age were excluded from the analysis.

**TABLE 1:** General characteristics of the participants of the included cohorts^*a*^

Cohort and characteristic	Young adults (ages 20–45 yr)		Middle-aged adults (ages 46–69 yr)^*b*^	
	Women	Men	Women	Men
AGP, U.S.				
No. of subjects	627	644	734	583
Age (yr)	34.60 (6.79)	33.76 (6.61)	56.13 (6.37)	57.13 (6.41)
SV richness	113.80 (33.04)	110.95 (31.51)	120.40 (0.89)	119.0 (0.78)
Shannon index	4.87 (0.83)	4.83 (0.80)	4.98 (0.89)	5.01 (0.78)
AGP, U.S. antibiotic consumers				
No. of subjects	136	83	147	91
Age (yr)	33.0 (7.75)	58.07 (6.56)	34.71 (6.87)	58.35 (6.52)
SV richness	100.38 (27.61)	97.80 (31.85)	107.07 (30.06)	108.71 (30.11)
Shannon index	4.64 (0.74)	4.70 (0.83)	4.72 (0.80)	4.83 (0.85)
AGP, U.K.				
No. of subjects	195	173	344	224
Age (yr)	35.90 (6.02)	36.40 (6.32)	56.45 (6.68)	57.75 (6.85)
SV richness	132.0 (31.69)	122.60 (32.38)	142.30 (36.27)	139.10 (36.23)
Shannon index	5.27 (0.69)	5.05 (0.92)	5.36 (0.83)	5.29 (0.80)
Chinese				
No. of subjects	946	670	1,826	1,521
Age (yr)	35.16 (6.73)	34.88 (7.07)	56.6 (6.59)	57.36 (6.75)
SV richness	101.80 (27.90)	99.66 (26.48)	99.41 (28.67)	101.4 (28.50)
Shannon index	4.47 (0.85)	4.40 (0.84)	4.36 (0.93)	4.35 (0.95)
Colombian				
No. of subjects	143	133	83	78
Age (yr)	33.83 (7.21)	34.21 (6.98)	52.48 (4.14)	52.90 (4.42)
SV richness	120.41 (30.21)	110.71 (31.06)	123.33 (32.75)	116.13 (33.95)
Shannon index	4.60 (1.05)	4.48 (1.12)	4.73 (0.99)	4.45 (1.13)
Cardiometabolic risk scale	−1.14 (3.07)	0.64 (3.67)	−0.36 (3.06)	1.39 (2.71)

a Values are given as the mean (SD).

b The ages of the Colombian individuals ranged from 20 to 62 years.

**FIG 1:**
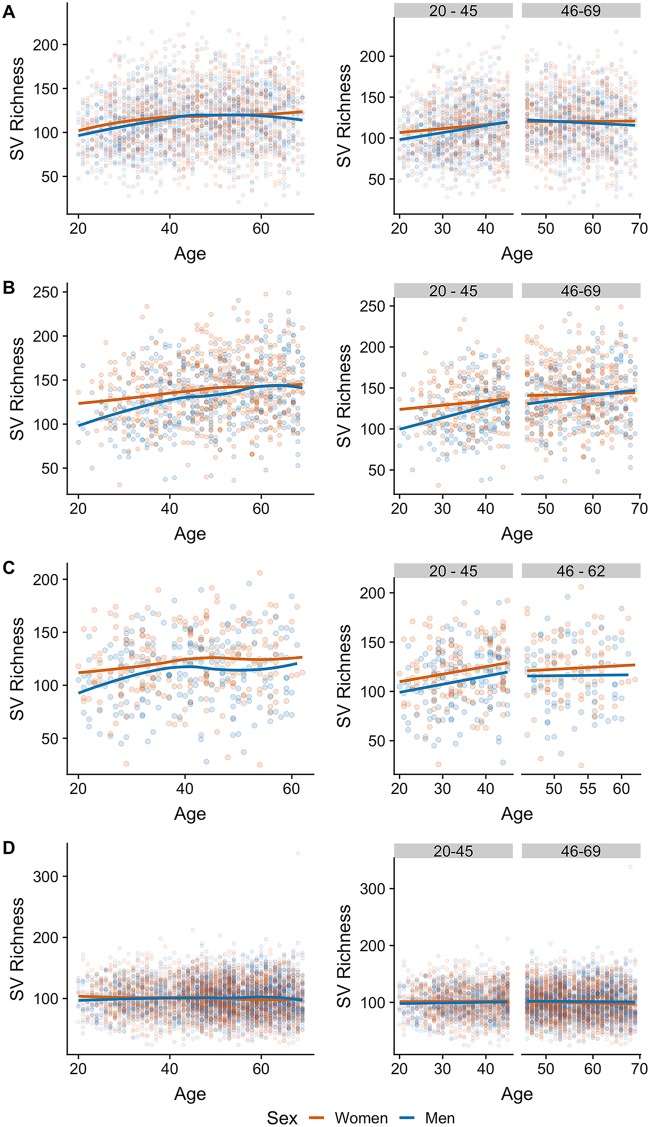
Gut microbiota richness is nonlinearly associated with age and differs between women and men in multiple populations: United States (*n* = 2,588) (A), United Kingdom (*n* = 936) (B), Colombia (*n* = 437) (C), and China (*n* = 4,963) (D). (Left) Sequence variant (SV) richness in adults ages 20 to 69 years (the age of the Colombians ranged from 20 to 62 years); lines indicate the relationship of richness with age after Loess smoothing for women and men separately. (Right) SV richness in young (age, 20 to 45 years) and middle-aged (age, 46 to 69 years) adults; lines indicate the linear regression fit for women and men separately.

To assess changes in alpha diversity with age during adulthood, we fit a simple linear regression model and a regression model with linear splines, in which the model is fit as two consecutive segments (20 to 45 years and 46 to 69 years; see Fig. S1 in the supplemental material). We then evaluated the goodness of fit of each model using the Akaike information criterion (AIC), which indicated that changes in alpha diversity are better explained by distinguishing between young adults (20 to 45 years of age) and middle-aged adults (46 to 69 years of age). In the U.S., U.K., and Colombian cohorts, we observed a positive but nonlinear association between alpha-diversity measures and age in both women and men. Loess curves fit independently by sex showed an inflection point after 40 years of age in each of these cohorts (Fig. 1A to C). In contrast, we did not observe such a pattern in the Chinese cohort, in which alpha diversity displayed a slight decrease with age (Fig. 1D).

10.1128/mSystems.00261-19.1 FIG S1 Linear spline regression of alpha diversity and age shows different associations in each age group in most populations. Lines represent the spline regression fit with a knot at 45 years. (Left) Sequence variant (SV) richness. (Right) Shannon index. (A) United States (*n* = 2,588); (B) United Kingdom (*n* = 936); (C) Colombia (*n* = 437); (D) China (*n* = 4,963). Download FIG S1, PDF file, 2.1 MB. Copyright © 2019 de la Cuesta-Zuluaga et al. 2019 de la Cuesta-Zuluaga et al. This content is distributed under the terms of the Creative Commons Attribution 4.0 International license.

Next, for each population, we fit linear regression models to examine associations of microbial diversity, age, and sex in each age group separately. In both the U.S. and U.K. cohorts, we observed a positive relationship between microbial richness and age for both sexes in young adults (adjusted *P* value [*P*-adjust], <0.001 for the U.S. cohort and <0.001 for the U.K. cohort), but not in middle-aged adults (*P*-adjust, 0.474 for the U.S. cohort and 0.216 for the U.K. cohort) (Fig. 1A and B). In addition, after accounting for age, differences in sequence variant (SV) richness tended to be higher in young adults (for the U.S. cohort, difference between men and women [Δ_men − women_] = −3.3 and *P*-adjust = 0.134; for the U.K. cohort, Δ_men − women_ = −9.84 and *P*-adjust = 0.024) than in middle-aged adults (for the U.S. cohort, Δ_men − women_ = −1.3 and *P*-adjust = 0.484; for the U.K. cohort, Δ_men − women_ = −3.7 and *P*-adjust = 0.270). Similar results were observed when we assessed taxon evenness using the Shannon index (Fig. S2). Similar to the U.S. and U.K. cohorts from the AGP, we identified a positive relationship between richness and age in the Colombian cohort in young adults of both sexes (*P*-adjust = 0.008) but not in middle-aged adults (*P*-adjust = 0.722) (Fig. 1C). Likewise, there was a difference in overall SV richness between the sexes in young adults (Δ_men − women_ = −10.0; *P*-adjust = 0.024) but not in middle-aged adults (Δ_men − women_ = −7.3; *P*-adjust = 0.225). In contrast to the U.S., U.K., and Colombian cohorts, we observed no association between microbiota alpha diversity and age in young-adult or middle-aged-adult Chinese (*P*-adjust > 0.1 for both comparisons) (Fig. 1D). Men in the Chinese cohort tended to have lower SV richness than women as young adults, yet the difference was not significant (for young adults, Δ_men − women_ = −2.14 and *P*-adjust = 0.194; for middle-aged adults, Δ_men − women_ = 2.04 and *P*-adjust = 0.107). We did not find evidence of an interaction between age and sex on microbial diversity in the studied cohorts for young or middle-aged adults with either of the diversity measures, after correcting for multiple comparisons (*P*-adjust > 0.15 in all cases). In all cohorts apart from the Chinese, the proportion of variance in alpha diversity explained by age and sex was moderate, yet it was consistently higher in younger adults than in middle-aged adults (Table S1).

10.1128/mSystems.00261-19.2 FIG S2 The patterns of the Shannon index are similar to those of SV richness in multiple populations: United States (*n* = 2,588) (A), United Kingdom (*n* = 936) (B), Colombia (*n* = 437) (C), and China (*n* = 4,963) (D). (Left) Shannon index in adults ages 20 to 69 years (the age of the Colombians ranged from 20 to 62 years); lines indicate the relationship of richness with age after Loess smoothing for women and men separately. (Right) Shannon index in young (age, 20 to 45 years) and middle-aged (age, 46 to 69 years) adults; lines indicate the linear regression fit for women and men separately. Download FIG S2, PDF file, 2.8 MB. Copyright © 2019 de la Cuesta-Zuluaga et al. 2019 de la Cuesta-Zuluaga et al. This content is distributed under the terms of the Creative Commons Attribution 4.0 International license.

10.1128/mSystems.00261-19.4 TABLE S1 Proportion of variance in alpha-diversity measures explained by age and sex in each age group from each population. *R*-squared values are from linear regression models. Download Table S1, DOCX file, 0.01 MB. Copyright © 2019 de la Cuesta-Zuluaga et al. 2019 de la Cuesta-Zuluaga et al. This content is distributed under the terms of the Creative Commons Attribution 4.0 International license.

**FIG 2:**
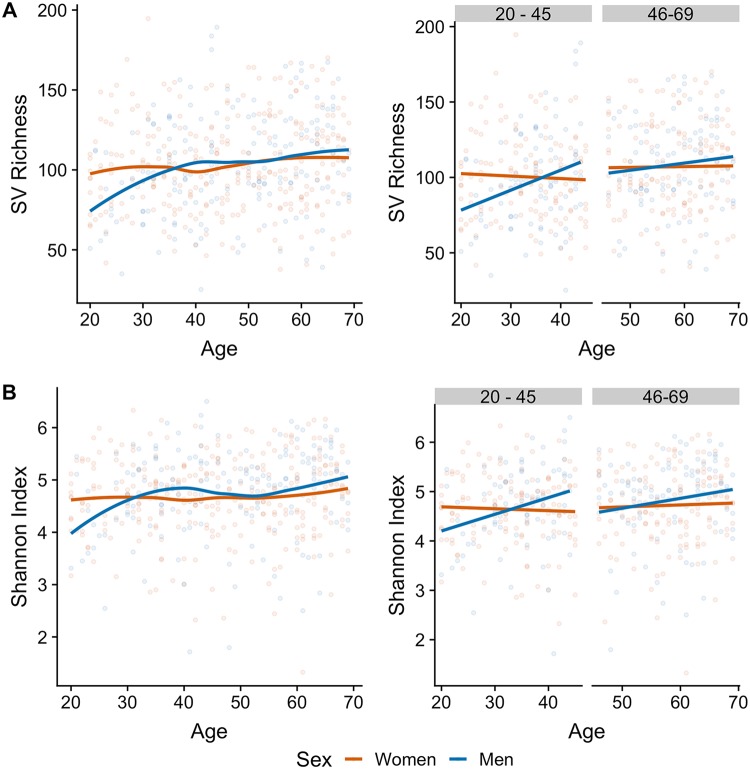
Antibiotic consumption has a limited association with the patterns of alpha diversity in U.S. adults that had consumed antibiotics 6 months prior to enrollment (*n* = 457). (A) SV richness; (B) Shannon index. (Left) Alpha-diversity metrics in women and men ages 20 to 62 years; lines indicate the relationships of richness with age after Loess smoothing. (Right) Alpha-diversity metrics in young (age, 20 to 45 years) and middle-aged (age, 46 to 69 years) adults; lines indicate the linear regression fit for women and men separately.

**FIG 3:**
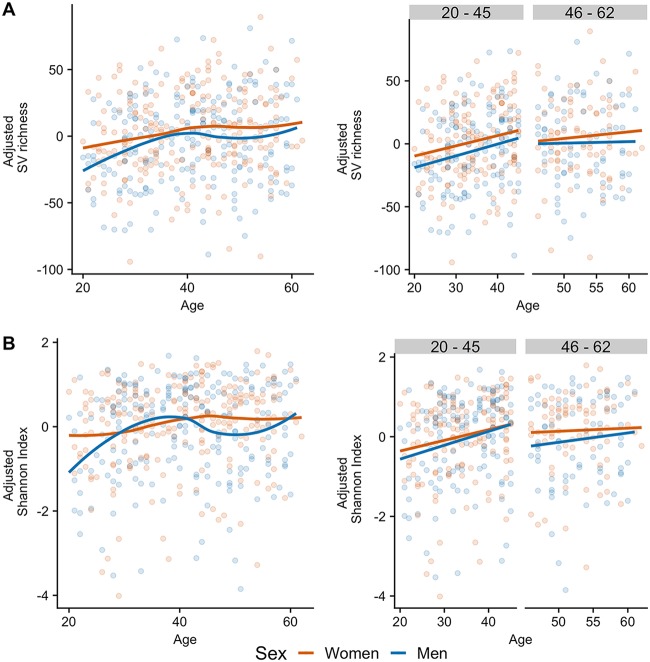
Adjusting alpha diversity by cardiometabolic health does not affect the observed patterns in Colombian adults (*n* = 437). (A) Residuals of SV richness; (B) residuals of the Shannon index. (Left) Adjusted alpha-diversity metrics in women and men ages 20 to 62 years; lines indicate the relationships of richness with age after Loess smoothing. (Right) Adjusted alpha-diversity metrics in young (age, 20 to 45 years) and middle-aged (age, 46 to 62 years) adults; lines indicate the linear regression fit for women and men separately.

Given that gut microbial diversity may be affected by factors such as antibiotic use or the cardiometabolic health of the host, we replicated the above-described analyses in cohorts in which we observed the patterns, making use of publicly available metadata. To test whether the consumption of antibiotics modified the observed pattern, we performed the above-described analyses on a set of 457 individuals (283 women and 174 men) from the U.S. cohort of the AGP that reported having consumed antibiotics in the 6 months prior to enrollment. We observed a lower SV richness in these individuals than in those that did not consume antibiotics (Table 1). Among the participants that consumed antibiotics in the past 6 months, we observed a similar tendency for alpha diversity to increase in the younger group and plateau in middle-aged individuals, with women having higher diversity than men, although there was a lack of statistical significance (Fig. 2). Likewise, we replicated the analyses in the Colombian cohort after introducing a composite measure of the cardiometabolic health of the subjects as a covariate into the linear models; after we adjusted the analyses for the cardiometabolic health score, the observed patterns were similar (Fig. 3).

To examine whether similar age- and sex-associated patterns would be observed when analyzing the relative taxon abundance in the gut microbiota, rather than using only alpha-diversity measures, we used a supervised machine-learning approach to compare the composition of the gut microbiota of the subjects of the different populations. We subdivided each cohort by sex, determined the SVs shared by both groups, and used their relative abundances and the chronological age at the time of sample collection of the host to fit a random forest (RF) regression model. Two models were built for women and men aged 20 to 69 years; each was trained using the data for one sex and tested on the other. For each subject, we calculated the relative microbiota age as the difference between its microbiota age and the microbiota age of the interpolated spline fit of an individual of the opposite sex at the same chronological age. Our results from random forest regressions indicated that the composition of the gut microbiota explained a low to moderate proportion of variance in chronological age, which varied by population and sex (Table S2).

10.1128/mSystems.00261-19.5 TABLE S2 Proportion of variance in random forest regression models of age by composition of the gut microbiota in each population. Download Table S2, DOCX file, 0.01 MB. Copyright © 2019 de la Cuesta-Zuluaga et al. 2019 de la Cuesta-Zuluaga et al. This content is distributed under the terms of the Creative Commons Attribution 4.0 International license.

**FIG 4:**
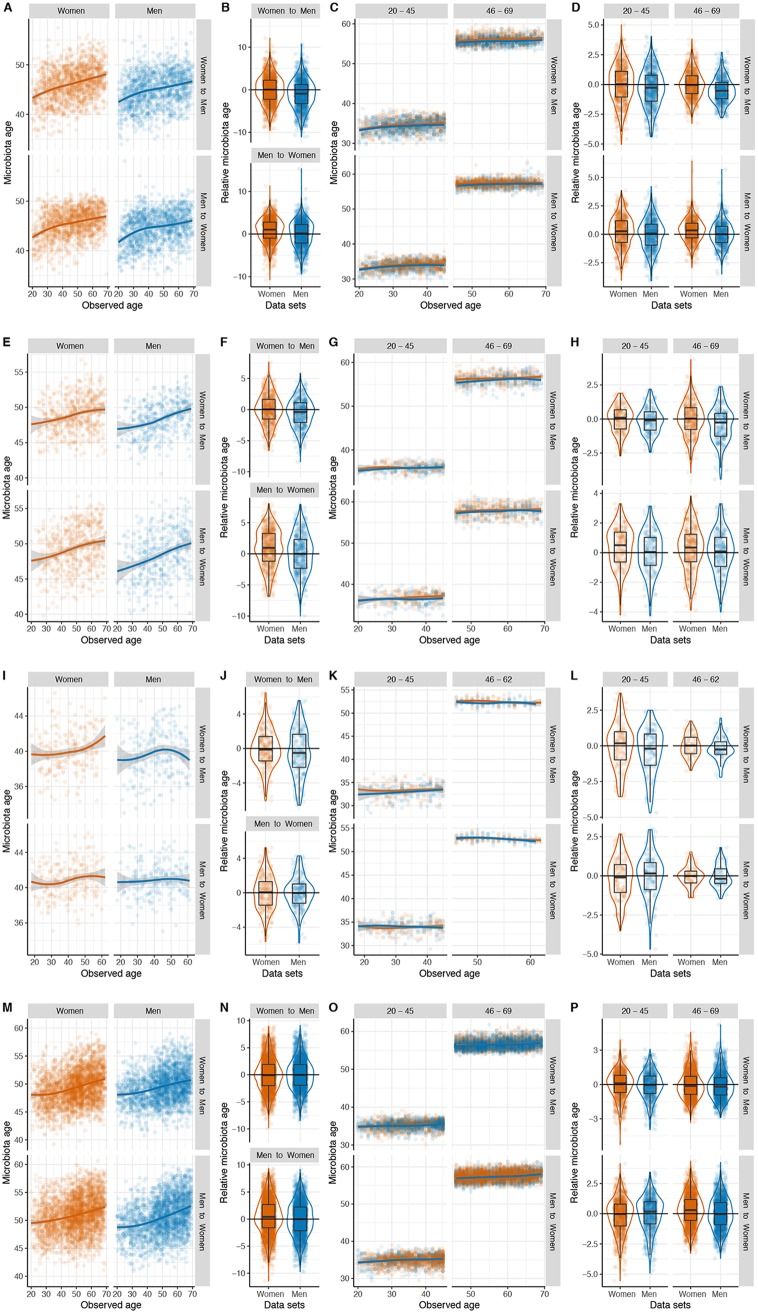
Gut microbiota age differs between women and men in multiple populations: United States (*n* = 2,588) (A to D), United Kingdom (*n* = 936) (E to H), Colombia (*n* = 437) (I to L), and China (*n* = 4,963) (M to P). For each population, the first set of panels (A, E, I, M) shows the microbiota age of women (orange) or men (blue), as calculated by a random forest (RF) model trained on the female (top scatter plots) or male (bottom scatter plots) subsets; lines indicate the spline fit. The second set of panels (B, F, J, N) shows the relative microbiota age (the difference of microbiota age of the interpolated spline fit based on the training data and microbiota age predicted in either training or test data) in women and men, which was derived from either an RF model trained on women and tested on men (top box plot) or an RF model trained on men and tested on women (bottom box plot). The third (C, G, K, O) and fourth (D, H, L, P) sets show results of analyses similar to those for the first two but are stratified by age group.

We used 1,494 shared SVs between women and men to build the RF model of the U.S. cohort (Fig. 4A). We found that men exhibited a lower relative microbiota age than women (in the women-to-men model, the difference between women and men [Δ_women − men_] = 0.81 years; *P*-adjust < 0.001, Wilcoxon rank-sum test; Fig. 4B, top), suggesting that sex is associated with the adult gut microbial aging process. To validate this finding, we also trained an RF model in the men and then applied it to the women (Fig. 4A, bottom); we found that women had a higher microbiota age (in the men-to-women model, Δ_women − men_ = 1.0 years and *P*-adjust < 0.001; Fig. 4B, bottom). To establish whether these trends were present in different age groups, we then examined the sex-dependent association of microbiota age in young and middle-aged adults separately. In the young-adult group, we selected the 1,311 shared SVs between both sexes to build the RF model for women and then applied it to predict the microbiota age of men. We found that young women exhibited a slightly higher relative microbiota age than men (Δ_women − men_ = 0.32 years, *P*-adjust < 0.001; Fig. 4C and D, top). Similar results were observed when we assessed the microbiota age in the middle-aged-adult group, in which we used the 1,601 shared SVs between sexes to build the RF model as described above. The microbiota age was higher in women than in men (Δ_women − men_ = 0.48 years, *P*-adjust < 0.001; Fig. 4C and D, top). Furthermore, such sex differences in microbiota age were not affected when we applied the men’s model to women (Fig. 4C and D, bottom). Likewise, in the U.K. cohort, we found that the microbiota age was higher in women than in men (women-to-men model, Δ_women − men_ = 0.51 years and *P*-adjust = 0.002; men-to-women model, Δ_women − men_ = 0.96 years and *P*-adjust < 0.001; Fig. 4E and F), using 1,613 SVs found in either the women’s or men’s microbiota for building and applying RF models. In addition, we observed significant or borderline significant differences in relative microbiota age between sexes in young adults (women-to-men model, Δ_women − men_ = 0.15 years and *P*-adjust = 0.186; men-to-women model, Δ_women − men_ = 0.36 years and *P*-adjust = 0.028; Fig. 4G and H) and middle-aged adults (women-to-men model, Δ_women − men_ = 0.41 years and *P*-adjust < 0.001; men-to-women model, Δ_women − men_ = 0.25 years and *P*-adjust < 0.091; Fig. 4G and H). In the Colombian cohort, we used 1,074 SVs shared between sexes to build the RF model; similar yet nonsignificant trends were observed between microbiota age and sex in the nonstratified analyses (women-to-men model, Δ_women − men_ = 0.38 years and *P*-adjust = 0.173; men-to-women model, Δ_women − men_ = 3.0e−05 years and *P*-adjust > 0.9; Fig. 4I and J) and in the young-adult group (women-to-men model, Δ_women − men_ = 0.18 years and *P*-adjust = 0.189; men-to-women model, Δ_women − men_ = 0.02 years and *P*-adjust = 0.292; Fig. 4K and L) and the middle-aged-adult group (women-to-men model, Δ_women − men_ = 0.27 years and *P*-adjust = 0.186; men-to-women model, Δ_women − men_ = 0.27 years and *P*-adjust = 0.873; Fig. 4K and L). We used 1,279 SVs shared between sexes to build the RF models in the Chinese cohort. The association between microbiota age and sex was not consistent when we cross-tested the models (women-to-men model, Δ_women − men_ = −0.07 years and *P*-adjust = 0.468; men-to-women model, Δ_women − men_ = 0.45 years and *P*-adjust < 0.001; Fig. 4M and N). We did not observe significant associations in the young-adult group (women-to-men model, Δ_women − men_ = 0.09 years and *P*-adjust = 0.183; men-to-women model, Δ_women − men_ = −0.17 years and *P*-adjust = 0.028; Fig. 4O and P), whereas in the middle-aged-adult group, we observed sex-dependent differences in microbiota age, and such differences tended to be consistent in the cross-application of the models (women-to-men model, Δ_women − men_ = 0.08 years and *P*-adjust = 0.059; men-to-women model, Δ_women − men_ = 0.31 years and *P*-adjust < 0.01; Fig. 4O and P).

Next, from the RF model trained on each sex to predict age from gut microbial composition, we determined the number of SVs that minimized the 10-fold cross-validation error of the models. We found that the error of the simplified models increased sharply when less than 500 SVs were used (Fig. S3). Finally, we obtained the taxonomic classification of the 500 SVs with the highest RF importance score in at least one of the models (Table S3). Overall, we found that SVs belonging to the families *Ruminococcaceae*, *Bifidobacteriaceae*, *Lachnospiraceae*, *Clostridiaceae*, and *Christensenellaceae* consistently had high RF importance scores, although the values differed between populations and within populations between men and women.

10.1128/mSystems.00261-19.3 FIG S3 Simplified random forest regression models of age by gut microbiota composition require approximately 500 SVs in all populations. Points represent the mean 10-fold cross-validation error of the random forest regression as a function of the number of SVs included, and bars represent standard deviations. Download FIG S3, PDF file, 0.5 MB. Copyright © 2019 de la Cuesta-Zuluaga et al. 2019 de la Cuesta-Zuluaga et al. This content is distributed under the terms of the Creative Commons Attribution 4.0 International license.

10.1128/mSystems.00261-19.6 TABLE S3 SVs with the highest random forest importance score in each population. The representative sequence of the SV, the complete taxonomic classification of the SV, and its corresponding importance score in each of the included populations and sexes are provided. Download Table S3, CSV file, 0.7 MB. Copyright © 2019 de la Cuesta-Zuluaga et al. 2019 de la Cuesta-Zuluaga et al. This content is distributed under the terms of the Creative Commons Attribution 4.0 International license.

## DISCUSSION

In this study, we analyzed the association of gut microbial alpha diversity with age and sex in three large cross-sectional cohorts encompassing four geographically distinct community-dwelling adult populations. Our analyses indicate that age is positively associated with gut bacterial diversity in men and women, with greater diversity being seen in women than in men. Notably, this association occurs in young but not middle-aged adults. Consistent with these findings, the predicted microbiota age varied based on sex, with stronger associations being seen in young adults. It is worth underscoring that while we did not observe these patterns in all studied cohorts, it was widespread and robust to technical differences, and the alpha-diversity shifts were not modified by the cardiometabolic health of the host or the antibiotic consumption in the cohorts for which this information was available. These findings provide new insights into the development of the human gut microbiome in adulthood according to both age and sex and emphasize the importance of including chronological age and sex as covariates in analyses of the human gut microbiota.

While the most dramatic change in gut microbiota diversity occurs in early childhood (7, 8), its increase in adulthood has also been reported (20, 21). In the cohorts in which the pattern was present, we observed an increase in alpha-diversity measures in young adults; however, this trend halted at about age 40 years (Fig. 1 and Fig. S1 in the supplemental material). This finding agrees with a previous report, in which no significant differences in alpha diversity were found between middle-aged adults and septuagenarians (21). The diversity of the gut microbiota continues changing after the seventh decade of life; it has been shown that centenarians have a higher alpha diversity than middle-aged adults, though it remains unknown whether this is the cause or the effect of healthy aging (20–22). However, gut microbiota diversity in the elderly can differ according to their community residence setting, as community dwellers have been shown to have a higher diversity than individuals in long-term residential care (23, 24).

Interestingly, the relationship between age and diversity was also linked with sex. Multiple studies have reported differences in the diversity and composition of the gut microbiota between female and male mice, which appear to be associated with a sex bias in the incidence of specific diseases, such as type 1 diabetes (12, 15), rheumatoid arthritis (14), and anxiety (25); sex-by-diet interactions have also been reported (26). While differences in alpha diversity between males and females were reported in humans and mice, we showed that the association between sex and alpha diversity was stronger in young adults than in middle-aged adults. In agreement with our results, no differences in alpha diversity were observed between women and men in a recent study in which the mean age of the participants was 60 years (27).

One of the most intriguing findings was the difference in gut microbiota richness between the sexes in young adults. This sex-dependent discrepancy suggests that women may enter adulthood with a more diverse gut microbiota, which plateaus at the same levels in both sexes by approximately age 40 years. The microbiota age models of young adults (ages 20 to 45 years) can explain about 2.5% more of the variance of chronologic age than those of middle-age adults. The establishment of different microbial communities in males and females may be mediated by sex hormones: female mice show a significant increase in alpha diversity during puberty (28), and differences in the composition of the microbiota increase with age but are eliminated by male castration (15). While little is known about the maturation of the human gut microbiota during puberty, we speculate that the differential hormonal milieu between women and men and the earlier timing of puberty in women may result in a more rapid diversification of the gut microbiota in women and that men only achieve the same level of diversification by middle age. Since our findings are based on cross-sectional data, future longitudinal studies are needed to disentangle age and birth cohort effects and the impact of factors such as steroid hormonal levels, pubertal transition, contraceptives, and lifestyle that may vary throughout life. Future research should also investigate specific microbial changes that may influence time-dependent sex differences on the biodiversity of the human gut microbiome.

While 3 of the 4 cohorts had an association between age, sex, and microbial alpha diversity, the Chinese cohort did not (Fig. 1 and Fig. S1), indicating that these associations are a widespread feature of the human gut microbiota whose universality remains an open question. The overall alpha diversity of this cohort, as measured by SV richness and the Shannon index, was lower than that of the other three cohorts. We also note that the exclusion criteria for this population were not the same as those for the populations in the other studies, with only a 1-month antibiotic exclusion and no stated exclusion of participants with diabetes or inflammatory bowel disease (18).

The striking similarity among the U.S., U.K., and Colombian cohorts with regard to age- and sex-dependent associations with microbial biodiversity arose despite the different geographical origins, sample sizes, and collection protocols of the studies. Moreover, we also found no apparent association of antibiotic use (U.S. or U.K. cohort; Fig. 2) or cardiometabolic health (Colombian cohort; Fig. 3) on the patterns observed in these cohorts, suggesting that the influence of age and sex on the microbiota may be similar in other ethnic and cultural groups beyond the influence of cardiometabolic disease and antibiotic consumption. Nevertheless, similar large-scale population studies should be performed or reanalyzed to determine the extent to which our results are generalizable to other populations, particularly in light of the findings for the Chinese cohort. Indeed, the contrast between the U.K., U.S., and Colombian cohorts and the Chinese cohort highlights the power of using large data sets and comparative analyses across cohorts to uncover subtle patterns and reveal novel insights not discernible in smaller studies. This is of critical importance, given the plausibility of population-specific disease signatures of the microbiome (18).

## MATERIALS AND METHODS

### Cohort description.

Fecal samples were obtained from individuals in three independent cohorts from four geographical locations. (i) The AGP data set is composed of two cohorts with individuals from the United Kingdom (539 women and 397 men) and the United States (1,361 women and 1,227 men) (Table 1) consisting of healthy participants with a self-reported age of between 20 and 69 years, a body mass index (BMI) of between 18.5 and 30 kg/m^2^, and no history of inflammatory bowel disease, diabetes, or antibiotic use in the past year. (ii) A cohort of Chinese individuals comprised 2,772 women and 2,191 men aged 20 to 69 years with a BMI ranging from 18.5 to 30 kg/m^2^ and no antibiotic consumption reported 1 month prior to fecal sample collection; pregnant women and hospitalized, disabled, or critically ill individuals were not included in the study. (iii) A cohort of community-dwelling Colombians (226 women and 211 men) consisted of individuals 20 to 62 years of age enrolled in similar proportions according to BMI, city of residence, and age range (20 to 40 and 41 to 62 years); underweight participants, pregnant women, individuals who had consumed antibiotics or antiparasitics in the 3 months prior to enrollment, and individuals diagnosed with neurodegenerative diseases, current or recent cancer (<1 year), and gastrointestinal diseases were excluded. Details on the data acquisition, quality assessment, and processing of fecal samples from these three cohorts were previously described (4, 18, 19).

### 16S rRNA gene sequence processing.

The amplicon sequences of all three cohorts were uniformly processed following the same procedures previously described (4). Briefly, the V4 hypervariable region of the 16S rRNA gene was sequenced with the Illumina MiSeq platform. Raw sequences were clustered into sequence variants (SV) with deblur denoising (29) using the QIIME 2 package (30). Sequence counts were rarefied to 1,250 reads per sample across all samples to mitigate uneven sequencing depth. Downstream analyses in the Chinese cohort were replicated using a rarefaction depth of 5,000 reads per sample, and 3,600 reads per sample were used in the Colombian cohort, to exclude the effect of rarefaction depth on alpha-diversity estimation (data not shown). Note, however, that the sample collection and DNA extraction methods differed between the studies.

### Statistical analyses.

SV richness and the Shannon index were calculated using QIIME 2, and statistical analyses were performed using R (v.3.4.3) software. The association of age and alpha diversity was measured with and without separate age groups by fitting linear models with linear splines (lspline [v.1.0] package of R) with a knot at the midpoint of the age range (45 years of age) and simple linear models, respectively. We assessed the goodness of fit of these models by means of the Akaike information criterion (AIC). Next, scatter plots of each alpha-diversity metric according to age were constructed, and then separate Loess curves for women and men were fit using the ggplot2 (v.3.0) package of R. Given the nonlinear association observed between alpha diversity and age, we subdivided the data sets into two separate age groups, 20 to 45 years (young adult) and 46 to 69 years (middle-aged adult), which were then used to fit linear models to test the associations of age (as a continuous variable) and alpha-diversity measures, stratified by sex; *P* values were adjusted for multiple comparisons using the Benjamini-Hochberg method (31).

Additionally, to account for the possible influence of participant antibiotic usage or cardiometabolic health on the observed associations, we conducted the following sensitivity analyses. For the former, we carried out the analyses using a separate group of individuals of the AGP cohort from the United States who had consumed antibiotics during the 6 months prior to their enrollment (283 women and 174 men). For the latter, we performed the analyses by adjusting the linear models for cardiometabolic risk in the Colombian cohort using a risk measure, which we termed the cardiometabolic risk scale (32). This was calculated using the sum of the z-scores of log-transformed waist circumference, triglyceride levels, insulin levels, diastolic blood pressure, and high-sensitivity C reactive protein levels; positive values of the score are associated with increased cardiometabolic health risk.

Random forest (RF) regression was used to regress the relative abundances of SVs in the gut microbiota of healthy women and men against their chronological age in each data set (randomForest R package of R) using the following parameters: ntree = 18,000 and mtry = *p*/3, where *p* is the number of input features (SVs). The microbiota age model was first trained on the training data set of female adults and was then applied to test the set of male adults, and vice versa. A smoothing spline function was fit between the microbiota age and the chronological age of the hosts for calculation of the relative microbiota age of the adults in the test sets to which the sparse model was applied. For a particular sample, the relative microbiota age was calculated as the difference between the microbiota age of a focal adult and the microbiota age of the interpolated spline fit of healthy female/male adults at the same chronological age. We further employed the Wilcoxon rank-sum test to compare the relative microbiota age between female and male groups in each data set. To determine the sex difference in microbiota age, we subdivided the data sets into the aforementioned age groups and repeated the analyses as described above in all age segments.

### Data availability.

Processed SV tables are publicly available via the Qiita QIIME database (Colombian study, accession number 11993; AGP study, accession number 10317; China study, accession number 11757). The code and data required to reproduce the statistical analyses are available at https://github.com/jacodela/microbio_aDiv.
