# Supplementary figures and images for: Age- and Sex-Dependent Patterns of Gut Microbial Diversity in Human Adults
Source: mSystems. 2019 May 14;4(4):e00261-19. doi: 10.1128/mSystems.00261-19 (PMC6517691; doi:10.1128/mSystems.00261-19)

**A**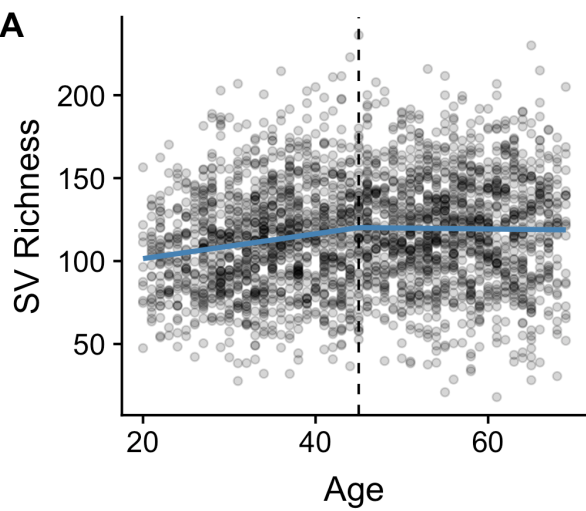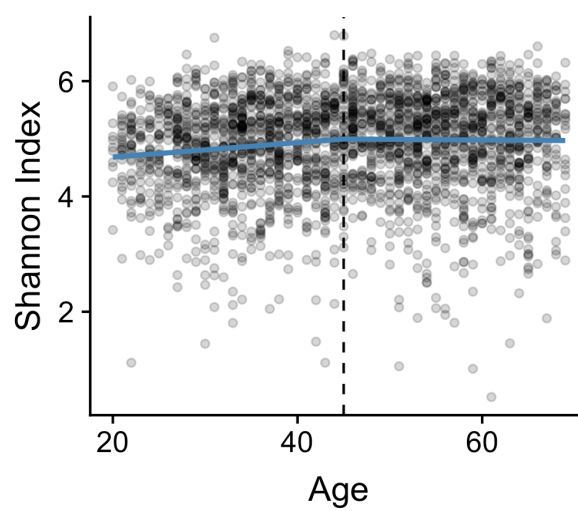**B**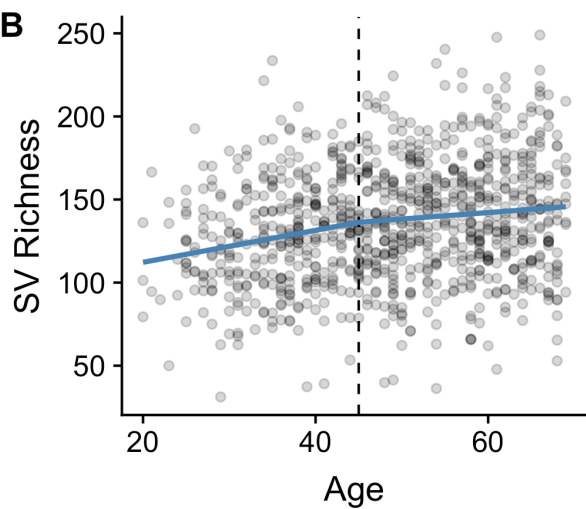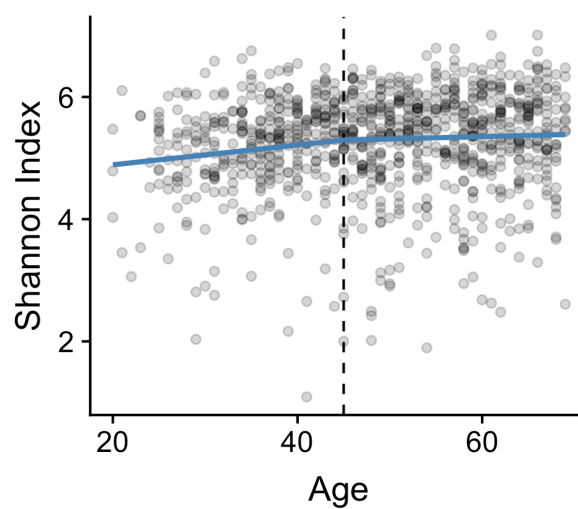**C**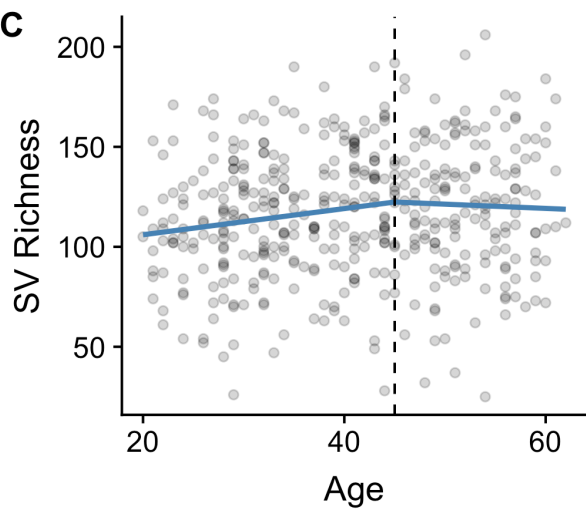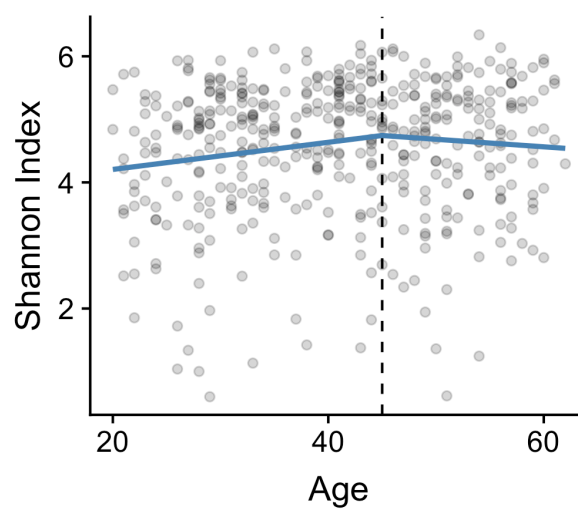**D**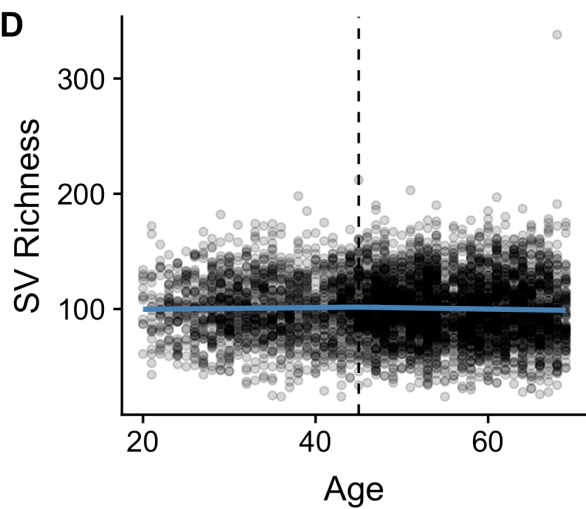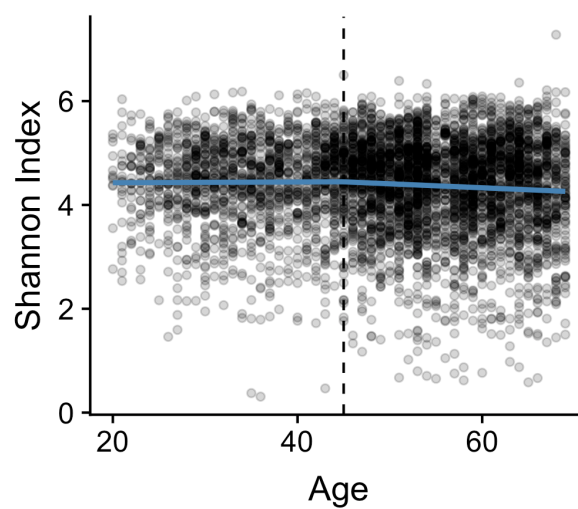

Supplement: FIG S1 [file mSystems.00261-19-sf001.pdf]

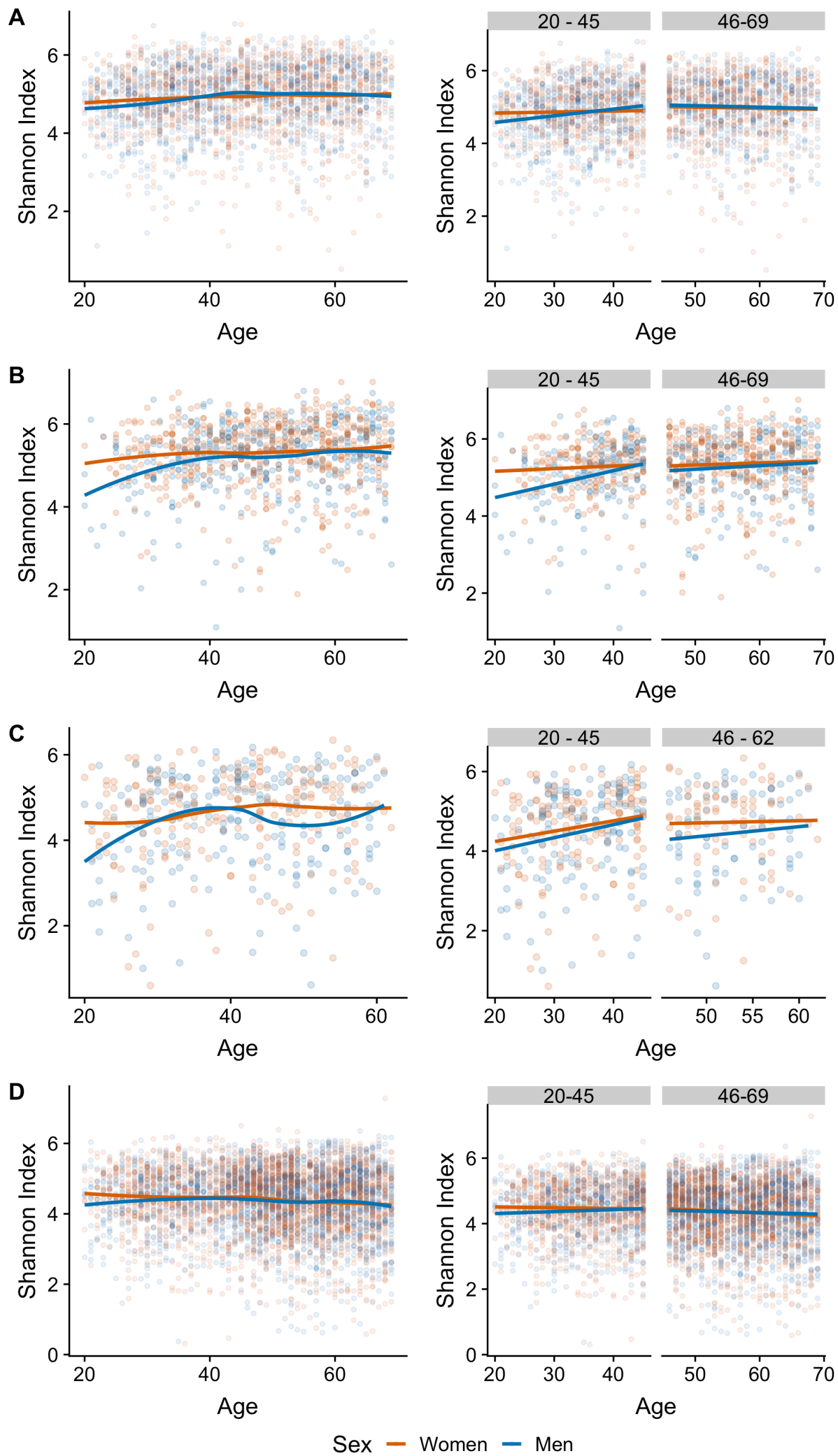

Supplement: FIG S2 [file mSystems.00261-19-sf002.pdf]

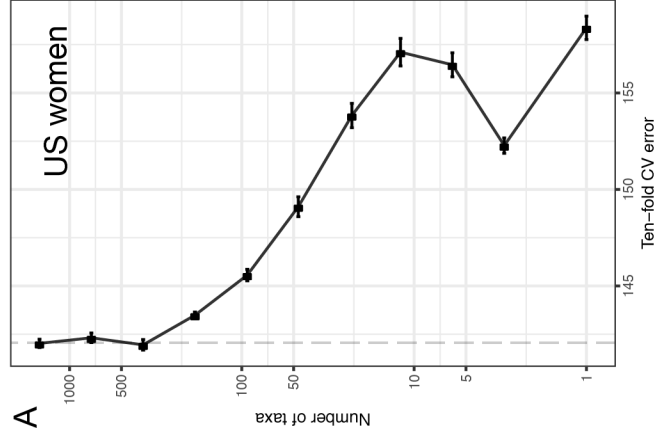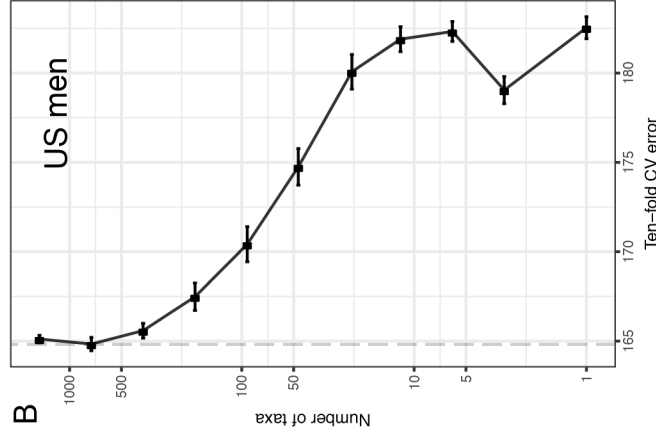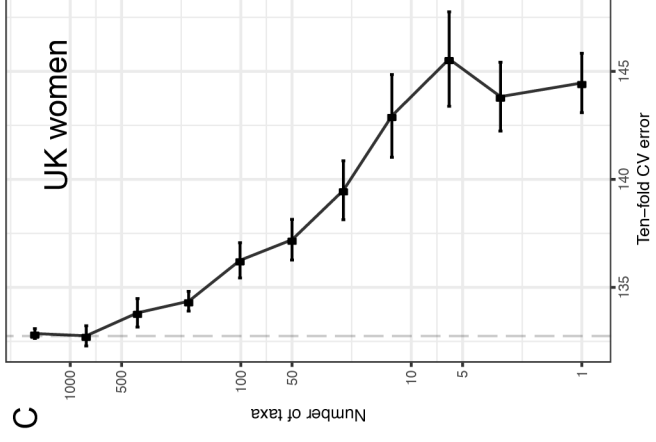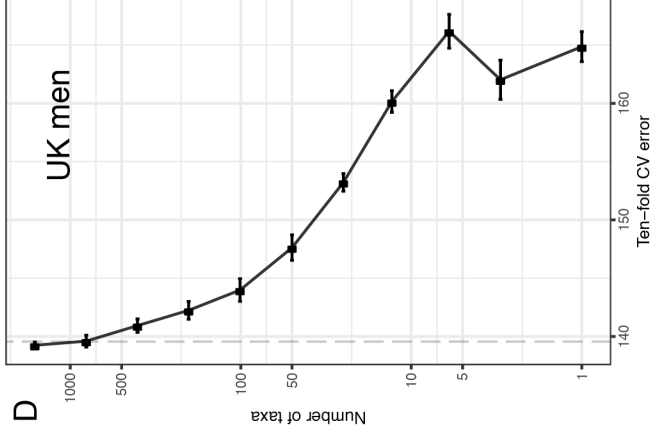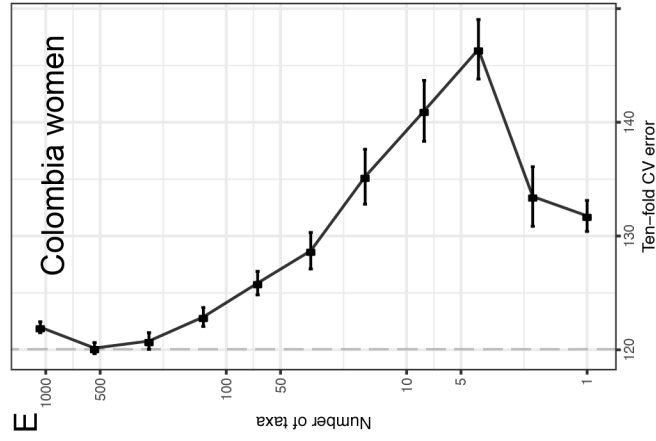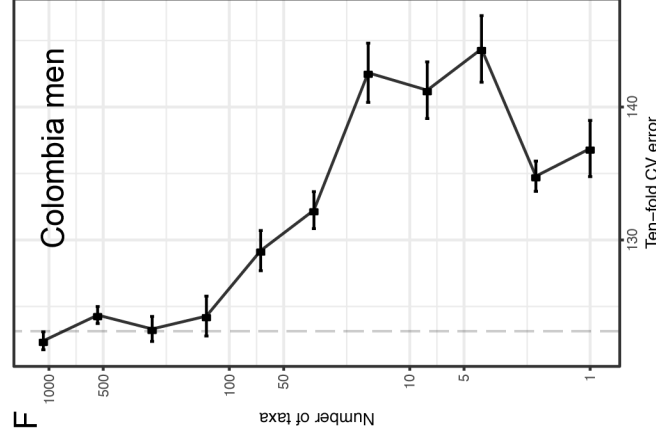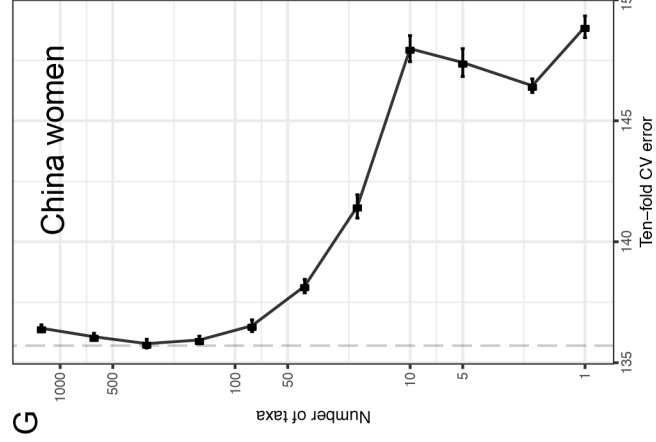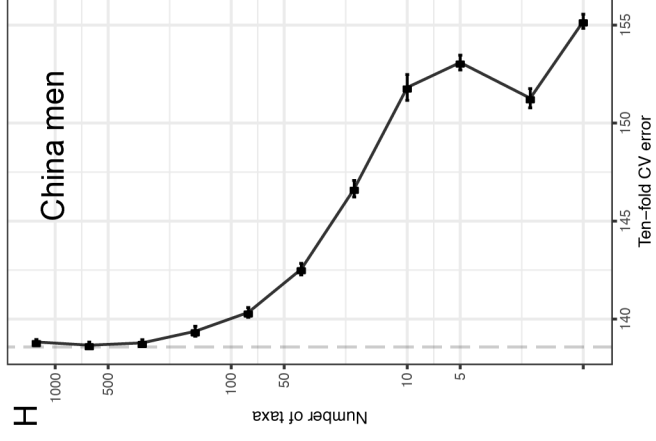

Supplement: FIG S3 [file mSystems.00261-19-sf003.pdf]
