# Supplementary material for: Age- and Sex-Dependent Patterns of Gut Microbial Diversity in Human Adults
Source: mSystems. 2019 May 14;4(4):e00261-19. doi: 10.1128/mSystems.00261-19 (PMC6517691; doi:10.1128/mSystems.00261-19)
Supplement: TABLE S1 [file mSystems.00261-19-st001.docx]

**Table S1**

|  | R-squared SV richness | | R-squared Shannon index | |
| --- | --- | --- | --- | --- |
|  | Young | Middle-aged | Young | Middle-aged |
| AGP-US | 0.023 | 0.001 | 0.008 | 0.001 |
| AGP-UK | 0.054 | 0.007 | 0.043 | 0.005 |
| Colombia | 0.057 | 0.012 | 0.040 | 0.020 |
| China | 0.002 | 0.002 | 0.002 | 0.003 |
