# Supplementary material for: Age- and Sex-Dependent Patterns of Gut Microbial Diversity in Human Adults
Source: mSystems. 2019 May 14;4(4):e00261-19. doi: 10.1128/mSystems.00261-19 (PMC6517691; doi:10.1128/mSystems.00261-19)
Supplement: TABLE S2 [file mSystems.00261-19-st002.docx]

**Table S2**

|  | Model | Pseudo R-squared |
| --- | --- | --- |
| AGP-US | Women | 0.1033978 |
|  | Men | 0.07440198 |
| AGP-UK | Women | 0.05947331 |
|  | Men | 0.1059874 |
| Colombia | Women | 0.03761303 |
|  | Men | -0.00243889 |
| China | Women | 0.0815143 |
|  | Men | 0.1075835 |
